# Supplementary figures and images for: Transcriptional Response in a Sepsis Mouse Model Reflects Transcriptional Response in Sepsis Patients
Source: Int J Mol Sci. 2022 Jan 13;23(2):821. doi: 10.3390/ijms23020821 (PMC8776114; doi:10.3390/ijms23020821)

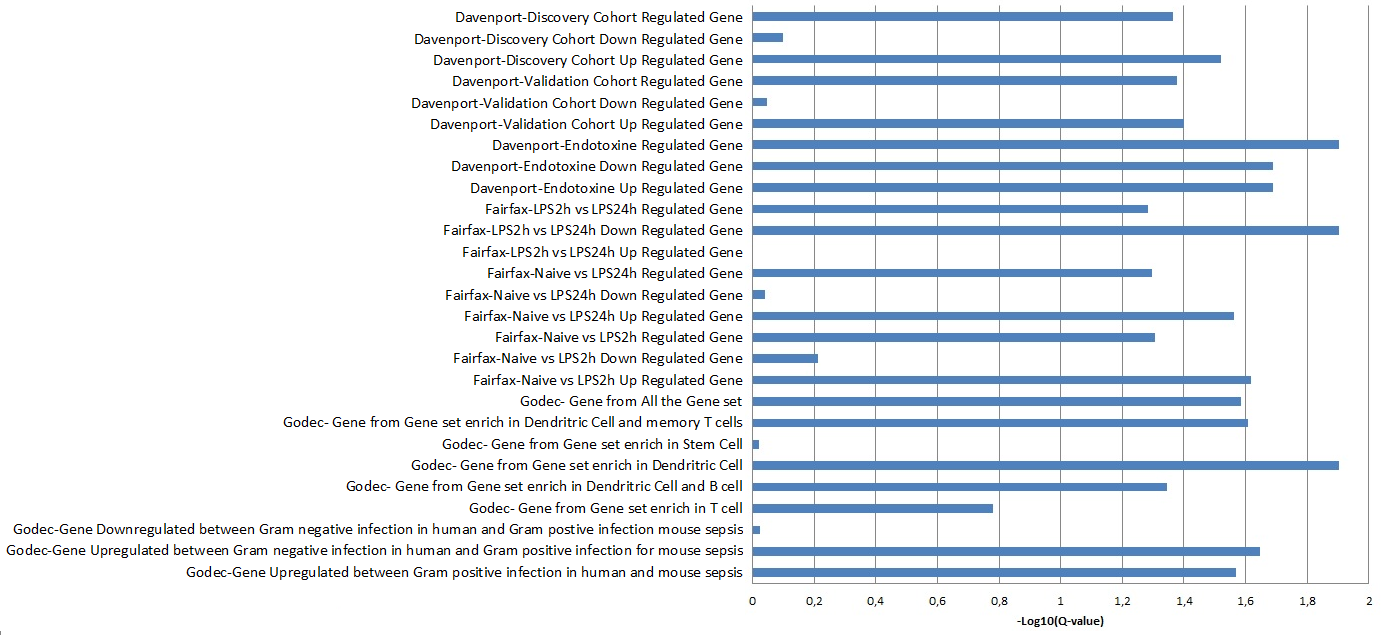

Supplement: Supplementary file 1 [file ijms-23-00821-s001.zip › Supplementary Figure S1.tiff]

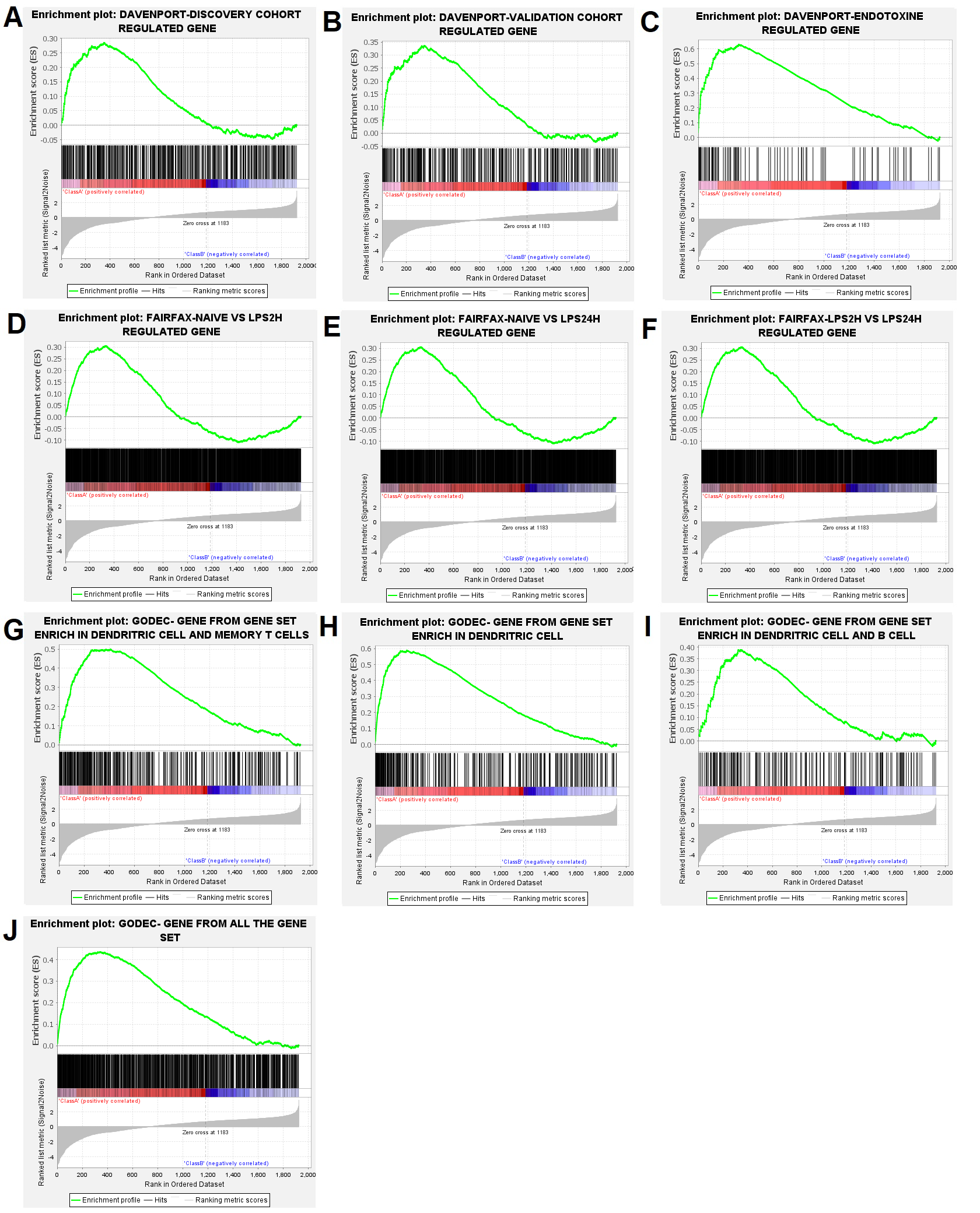

Supplement: Supplementary file 1 [file ijms-23-00821-s001.zip › Supplementary Figure S2.tiff]
